# Themes arising during implementation consultation with teams applying family-based treatment: a qualitative study

**DOI:** 10.1186/s40337-018-0218-y

**Published:** 2018-11-01

**Authors:** Jennifer Couturier, Melissa Kimber, Melanie Barwick, Tracy Woodford, Gail McVey, Sheri Findlay, Cheryl Webb, Alison Niccols, James Lock

**Affiliations:** 1 Department of Psychiatry and Behavioural Neurosciences, McMaster Children’s Hospital, McMaster University, 1200 Main St W, Hamilton, L8N 3Z5 Canada; 2 Offord Centre for Child Studies Suite 201A, McMaster University, 175 Longwood Rd S, Hamilton, L8P 0A1 Canada; 3 Research Institute, The Hospital for Sick Children, 555 University Avenue, Toronto, M5G 1X8 Canada; 4 Department of Psychiatry and Behavioural Neurosciences, McMaster University, 1200 Main St W, Hamilton, L8N 3Z5 Canada; 5 Toronto General Hospital Research Institute, University Health Network, 200 Elizabeth St, Toronto, M5G 2C4 Canada; 6 Department of Pediatrics, McMaster Children’s Hospital, McMaster University, 1200 Main St W, Hamilton, L8N 3Z5 Canada; 7 McMaster Children’s Hospital, 1200 Main St W, Hamilton, L8N 3Z5 Canada; 8 Department of Psychiatry & Neurosciences, Stanford University, Stanford, 401 Quarry Rd, Palo Alto, CA 94304 USA

**Keywords:** Family-based treatment, Implementation, Children, Adolescents, Consultation

## Abstract

**Background:**

This study describes themes arising during implementation consultation with teams providing Family-Based Treatment (FBT) to adolescents with eating disorders.

**Methods:**

Participants were implementation teams (one lead therapist, one medical practitioner and one administrator) at four sites. These teams agreed to support the implementation of FBT, and participated in monthly consultation calls which were audio-recorded, transcribed verbatim and coded for themes. Twenty percent of the transcripts were double-coded to ensure consistency. Fundamental qualitative description guided the sampling and data collection.

**Results:**

Twenty-five (average per site = 6) transcripts were coded using thematic content analysis. Six major themes emerged: 1) system barriers and facilitators 2) the role of the medical practitioner, 3) research implementation, 4) appropriate cases, 5) communication, and 6) program impact.

**Conclusions:**

Implementation themes aligned with previous research examining the adoption of FBT, and provide additional insight for clinical programs seeking to implement FBT, emphasizing the importance of role clarity, and team communication.

## Plain English summary

Many challenges can arise when treatments that have been tested in academic settings are transferred to “real life” clinical settings. This study attempted to examine what barriers arose while teams were attempting to integrate Family-Based Treatment for adolescents with eating disorders into their programs. The main issues that arose for teams of administrators, medical practitioners and therapists involved: 1) system barriers, 2) the role of the medical practitioner, 3) the implementation of the research component of the study, 4) finding appropriate cases, and 5) communication. Despite these challenges, teams mentioned a positive program impact of the implementation of Family-Based Treatment.

## Background

Despite the evidence suggesting that Family-Based Treatment (FBT) is effective in treating children and adolescents with eating disorders, and has the potential to reduce treatment costs by up to 70% [1], research indicates that few therapists consistently use this model, or if they do, it is not practiced with fidelity [2]. Fidelity to a treatment model is important with respect to replicating outcomes from research trials [3]. Thus, the need to test and evaluate contextually appropriate implementation strategies to promote the uptake and implementation of FBT with fidelity is a necessary endeavour. It is important here to differentiate the terms Evidence-Based Practice (EBP) versus Evidence-Based Treatment (EBT); the former being a clinical practice which incorporates evidence considering the context as well as clinician and patient preferences, while EBT refers to a treatment applied with fidelity. For the purposes of this paper, we will be focused on the implementation of FBT with fidelity; therefore, as an EBT.

Eating disorders are among the most pernicious psychiatric disorders afflicting adolescents; they frequently run a chronic and relapsing course and can have longstanding impacts on all aspects of health [4]. Equally concerning is that few adolescents living with an eating disorder come to the attention of specialized mental health services, and those that do may not receive evidence-based interventions [5]. A cross-sectional survey of clinicians (*n* = 117) utilizing FBT in their practice indicated that they rarely implement all intervention elements with their adolescent patients and that they utilized the intervention model with patients that were older than what is recommended [6].

Qualitative research with clinicians (*n* = 40) and program administrators (n = 11) suggests that variation in FBT use may be a function of clinician, patient and program factors. Nineteen of the 40 therapists interviewed reported having received training in the FBT model, with 31 of the 40 therapists reporting that they had read some or the entire FBT manual. However, none of the therapists practiced the model with fidelity and they reported elevated levels of interpersonal anxiety when attempting to implement certain model elements, including the family meal, weighing the patient, and limiting the involvement of the dietician in the treatment process [2]. They also voiced concern about the application of the treatment with adolescents with multiple co-morbidities [2]. Critically, all of the clinicians acknowledged the importance of having administrator support for implementation of the evidence-based treatment, and the program administrators (*n* = 11) reported that all clinicians within their respective programs would require further training *and* ongoing supervision in order to deliver FBT with fidelity [7].

In terms of implementation models, a number of frameworks have been published, however, many of these approaches and models have shown inadequate implementation effectiveness overall [8–10]. A major contribution to the field of implementation science has been the Active Implementation Frameworks (AIF) from the National Implementation Research Network (NIRN) [9], which characterize the overarching process of implementation, and have been used to inform the successful adoption and implementation of Evidence-Based Treatments (EBTs) within the mental health sector [11–16]. A crucial component of the AIF is the use of implementation teams. The role of the implementation team is to oversee and monitor the implementation process and devise procedures and protocols to support the implementation of the EBT within everyday practice. According to Fixsen et al. [9], an implementation team would consist of four or five individuals, who represent core areas of the organization or program, are familiar with the EBT, as well as the program and organizational processes influencing the use of the EBT. Recent research suggests that involvement of EBT trainers on the team is facilitative [13]. The implementation team has been identified as a seminal component to the change endeavour and a compliment to other implementation drivers [9, 11–13, 17, 18].

While applications of the AIF in criminal justice, addictions [19] and child welfare [20–22] provide insight into its utility as an over-arching framework for EBT implementation, a single large-scale case-study testing the field use of the AIF in child and youth mental health services in Canada provides promising evidence for its use in pediatric eating disorder treatment services. In their effort to shift all treatment programs to EBTs, Kinark Child and Family Services partnered with Implementation Science Researchers at The Hospital for Sick Children and used NIRNS’s AIF to inform their implementation intervention. Specifically, Kinark adapted the AIF to their organizational context and managed to effectively adopt and implement eight EBTs across their organization over four years of study [12, 13].

Due to the paucity of implementation literature in the field of eating disorders and even more specifically for children and adolescents, the following study was designed to evaluate the extent to which an adapted AIF could support the uptake, implementation and sustainability of FBT within four pediatric eating disorder treatment programs in Ontario, Canada. Based on the available evidence for the AIF, our implementation model included: 1) the establishment of implementation teams, 2) a training workshop, 3) monthly clinical consultation, 4) monthly implementation consultation and, 5) fidelity assessment. The objective of this paper is to identify and describe themes arising in implementation consultation component of the model.

## Methods

### Design

Data for this study come from a larger, multi-site (*n* = 4), mixed method, pre-post FBT implementation study. The methods for this study are described in another publication in which we report on findings from the clinical consultation component of this study [23]. Briefly, informed by an NIRN’s AIF [24] our larger study purposefully recruited therapists, physicians and administrators in four Ontario-based pediatric eating disorder programs to undergo training, clinical consultation in the FBT model, and explored implementation processes and participant experiences of these processes. Each of the participating organizations was asked to identify an implementation team that consisted of an administrator/manager, a lead therapist and a physician who would be charged with supporting FBT training, supervision, implementation and research processes for this study. In addition, the implementation team was asked to identify therapists in their program who were most appropriate and willing to undergo training in the FBT model. The administrators and physicians did not participate in the clinical consultation reported elsewhere [23].

### The treatment model

The treatment model used for this study involved a standard FBT protocol described in Couturier et al. [23]. The FBT manual was used to train clinicians in the model [25]. Family Based Treatment is an outpatient, intensive treatment in which the family is the primary resource to re-nourish the affected child [25]. FBT involves three phases of treatment over 9 to 12 months. The first phase focuses on helping the family to restore the child’s weight and interrupt disordered eating behavior. The second phase involves the transition of control over eating behavior back to the adolescent. The third and final phase addresses developmental issues such as physical development, peers and dating, and separation and individuation.

### Study procedures and data collection

Implementation issues were captured during separate monthly phone calls with each of the implementation teams (consisting of a therapist, administrator and medical practitioner), co-led by FBT (JC) and implementation (MK) experts. Implementation consultation calls were audio recorded, and transcribed verbatim for qualitative data analysis, differentiating the comments of the lead therapists, medical practitioners, administrators and the consultants. The audio recording and transcription of implementation consultation calls were guided by the principles of fundamental qualitative description [26]. As opposed to clinical consultation calls which focused on clinical issues with respect to the content and process of FBT sessions involving the therapists [23], the implementation calls focused on any practical issues related to the implementation of FBT at each site, and involved implementation team members.

This study received ethical approval from the Hamilton Health Sciences/McMaster Faculty of Health Sciences Research Ethics Board as well as the ethics boards at all participating sites.

### Data analysis

Our data analysis methods are described elsewhere [23]. In brief, conventional content analysis [27] was used to guide first and second levels of coding. Key concepts were identified through line-by-line coding. A codebook was generated and refined through multiple readings of the transcripts, in consultation with the research team, as well as through the process of theoretical memoing [28]. All transcripts were coded by an experienced qualitative data coder (TW), and 20% of these transcripts were independently double-coded by the principal investigator (JC). A third team member (MK) resolved any disagreements through consensus. Summative content analysis was used to provide counts of codes [27]. For specifics regarding the methods used for the qualitative data analysis please see Couturier et al. [23]. Coding was completed using Nvivo 10 (QSR International Pty Ltd., Version 8, 2008).

## Results

### Descriptive data

Implementation teams consisted of administrators, therapists and medical practitioners as described above. The twelve participants included two males and ten females with an average age of 46.7 + 10.5 years (range 28 to 60 years). Participants had been in their current role for an average of 7.9 + 7.0 years (range 1 month to 25 years). Twenty-five (average per site = 6, range 4 to 9) implementation consultation calls were completed over a period of 9.5 months (range 7 to 12 months). Attendance on the calls was as follows: lead therapist 96% (24/25), medical practitioners 48% (12/25), administrator 64% (16/25). The number of implementation consultation calls was 25 with an average of six sessions per site (range four to nine). The calls ranged in length of time from 15 min to 52 min with an average of 27 min (SD = 9 min).

### Implementation themes

Table 1 outlines the six major themes that emerged: 1) system barriers and facilitators 2) the role of the medical practitioner, 3) research implementation, 4) appropriate cases, 5) communication, and 6) program impact.

**Table 1:** Implementation Themes

Theme	Sources	References
System Barriers and Facilitators	21	200
Medical Role	20	106
Research Implementation	18	62
Appropriate Cases	15	77
Communication	14	79
Program Impact	4	26

In terms of **system barriers and facilitators**, implementation team participants mentioned several different types of barriers to enrolling families into the FBT study. These included having lengthy waitlists, not having enough clinicians to pick up cases quickly enough, lack of dedicated time for the medical practitioner to monitor children in their programs, and having a central intake process that was independent from their program (as this resulted in inappropriate referrals at times). The Central intake process is identified as a barrier in this quote:

*There's an 11 year old who's on the inpatient unit and “physician’s name” is trying to get her to see us but they have to go through Central Intake (first). We've got some of those issues.*

In relation to system barriers for implementation, one lead therapist shared the following about how their waitlist impacted on enrolling families in FBT and our implementation study:

*Oh really, honestly I think the only thing really is our wait list? Cause you know, some of the kids that I'm looking, at I think "Oh some of these would be really great for FBT." But they're a little ways away [geographically] or they end up hospitalized by the time they get here.*

With respect to the **role of the medical practitioner**, the overlap with the role of the therapist was a topic discussed frequently by implementation teams. Some medical practitioners were unsure which topics they were allowed to cover in their medical check-ins according to manualized FBT and were cognizant that they did not wish to overstep into the territory of the therapist. This concept was illustrated here by a medical practitioner:

*I think that there's overlap in terms of what I’m doing and what I’m saying. You know, I just feel like from a pure manualized FBT …. I'm wearing some of the therapist's hat.*

Further role challenges are illustrated in the following description from a medical practitioner on the team:

*Well it's a bit, it's less, I' mean it's fine, but it's less straight-forward because... And I even had one of my trainees ask me yesterday in clinic, why my role was different between patients? You know she was like... Cause you know, it was an FBT case? They're both FBT cases but one is a, you know, it's a manualized case, and so you know, I’m just staying away from anything that relates to nutrition.*

The understanding of the medical practitioner role seemed to evolve over time with the consultation provided. Early on in the study one practitioner mentioned the following:

*Practitioner: I would say from a job satisfaction standpoint, I would say, my job satisfaction would be, kids that are in the study is far lower than it is in kids that aren't in the study.*

*Consultant: How come?*

*Practitioner: Just because I feel like um, I'm not really doing anything. I'm just looking at their heart rate, checking their blood pressure. It's like I feel like I don’t really have the same level of engagement or bond that I had with the patients that are prior.*

*Consultant: Okay. So, you feel like your hands are tied a bit?*

*Practitioner: Totally. But like I don't, and to be perfectly honest, I don't feel, like I feel like they're getting, that the care that they're getting is not as good as the care they were getting before.*

After several months of consultation the same medical practitioner voiced the following:

*Practitioner: Yeah, I know, I think it hindsight? I think a lot of the confusion that I had, really was a, a lot of the concerns I had were unfounded because I think the reality is, you know, 95% of what I was doing was completely what I am still doing. The only, I mean, I think you provided clarity I think on one phone call and you were like "The only thing that you really gotta to stay away from involves the, you know, specifically guiding the therapy as it relates to weight gain".*

Implementation teams discussed the difficulty with the caseload of the medical practitioner and lack of time to discuss cases. They discussed practical issues such as who should weigh the patient first, the medical practitioner or the therapist. They also discussed the frequency of visits with the medical practitioner and whether the visits were too frequent or not frequent enough. At some sites, the medical practitioner picked up cases before the therapist due to the longer wait time for the therapist, creating a situation where FBT was started by the medical practitioner and carried forward by the therapist.

Implementation teams discussed several challenges with respect to the **implementation of our research protocol**. They were challenged by the time involved in completing fidelity measures and how to obtain these measures from families in a nonbiased way. The time and effort involved in faxing the measures and sending audio-recorded files electronically was also a barrier. They experienced problems with recruitment of families, and discussed how to gain consent from families in a way that would not involve any external pressure. One team decided to have the administrator gain consent from families, as she would not be providing treatment. One administrator spoke about the difficulties with recruitment in the following way:

*And my understanding is both declined the research project but they are still continuing with FBT…My understanding is that part of the reason was the tape recorder issue; they didn't want to be taped.*

Teams mentioned a few areas for improvement in future research. They thought that videotaping would be better than audiotaping, as more information can be captured related to body language and facial expressions, which are very important in any type of psychotherapy. This idea is articulated by this therapist:

*Therapist: The only thing I would say is, would be useful is for you to actually see the sessions live, not live, but on tape. Then, then what you see would actually be useful.*

*Consultant: Uh hum, you think the video would be that much more impactful than the audio?*

*Therapist: Yep. Frankly I think you made a mistake by just doing audio.*

Another suggestion was to view sessions in real time over videoconference, especially the family meal session. Teams also mentioned that having additional time to participate in the research study would be helpful, particularly for completing administrative tasks such as faxing and electronically sending data.

During the initial implementation phases, implementation teams frequently discussed the challenges of recruiting **appropriate cases**. Psychiatric co-morbidity was high among their patients waiting for treatment, and they wondered if they would be good first cases in which to implement FBT given that they wanted to adhere strictly to the manual. This concept is illustrated by this therapist:

*I don’t know whether you guys are experiencing this too but the co-occurring stuff and just the complexity of the family dynamics and the parents are often, more mentally, uh, challenged with their own stuff than the kids are. So how do you do pure anything these days?*

They mentioned challenges with parental psychopathology and whether these parents could manage the demands of FBT. One therapist mentioned the following with respect to these issues:

*I mean that's been our pattern in terms of pick-up. You know, it's hit and miss. We will get clients who I think will be really appropriate for FBT but we also have a number of clients who you know may not fit that criteria because either you know, parents are not well, you know well enough, there's some pathology going on with parents, you know there's just a lot of dysregulation and it's just not gonna work. In terms of pick-up that's been our issue.*

Teams voiced a reluctance to implement FBT among cases for whom binge eating and purging were part of the symptom constellation for fear that this could reduce the effectiveness of the treatment. They also mentioned the need for cases to be medically stable in order to participate. The concerns regarding complexity and severity are illustrated by this administrator:

*I think really what it is that the kids that are being referred to us are already so complex? They're often have already been in hospital, they’re often from families where families are not intact, and we’re not seeing the early presentations. We're not seeing typically, we're not seeing kids where this is in the first year or so. Typically, by the time they're being referred to us that a lot of them would be definitely day treatment if not inpatient requirement for the kind of treatment they need.*

**Communication** issues were discussed throughout the study period. Teams indicated there was little time to discuss FBT cases outside the implementation and clinical consultation phone calls because their teams did not have weekly clinical rounds where outpatient care could be discussed. One lead therapist outlined these challenges with communication here:

*We have a couple of rounds, we have an inpatient team rounds and we have a day program team rounds, but there's no formal outpatient rounds….But I do think from the, you know, from therapists' perspective, myself and my colleagues, we work very hard with the outpatient cases because they don't get talked about in rounds and because they're seen in clinic and we don’t' always know what's going on. So we work hard to liaise with the physicians on those cases when we have concerns.*

Relatedly, therapists were challenged by the scheduled rotation of medical practitioners because it prohibited their ability to meet and discuss FBT with specific cases on a regular basis. One therapist had this to say:

*Just cause they're always rotating and we can't always, you know we try to get the same pediatrician but sometimes it rotates. So that's been a big challenge for us with doing FBT.*

Teams discussed how the FBT implementation study impacted the program (**program impact**). They perceived a trickle-down effect on clinical practice manifested by the curiosity of medical personnel not directly involved in the study. There was also interest from front line inpatient staff who asked about the study when patients were discharged sooner than expected. One therapist indicated the following:

*I think it's had a great trickledown effect, even for those therapists who aren't officially in this study. Um, the taking on cases that we are trying manualized and showing some other very positive results and, I don’t know. I just, uh I feel like it's, it's a study that's had a lot of impact on how we deliver services.*

Some team members mentioned a reduction in readmission rates, faster discharge from hospital, and improved outcomes for patients. One medical practitioner illustrates this point:

*I know 100% our hospitalization admission rate has decreased, and I know 100% that our length of stay has decreased. We are admitting people less. And when we are admitting and the biggest, the biggest difference I think would be felt in the off-service patients. So you know, historically, I don't know what the numbers are but I can tell you for sure we ran on average, anywhere from 3 to 7 patients off-service. The average was probably the mean, was probably around 3. But I'd be, I’m sure that the mean is less than that now. Definitely less than that. And the mean, if is not significantly, if the mean hasn't been significantly affected, the length of stay has definitely been affected.*

## Discussion

This is the first study to use implementation team methodology within the eating disorders field, although it has been used successfully elsewhere to support EBT implementation in children’s mental health [12, 13]. As such, it was important to capture themes arising during the FBT implementation process using qualitative methodology to thoroughly understand therapist, medical practitioner and administrator perspectives of the components related to the implementation process. Outcomes with respect to treatment fidelity and post-study satisfaction will be reported in a subsequent paper. Many of the themes identified in this study support those identified in our previous work with therapists and administrators on their views of FBT uptake [2, 7]. The current study captures clinician and administrator perspectives during an actual period of implementation whereas our previous work examined their views prior to an implementation project. In addition, no prior work has explored the perspectives of medical practitioners with respect to FBT implementation.

System barriers previously identified as impacting FBT uptake [2] emerged again as significant issues in this study. Managing waiting lists, lack of resources, and time management were identified as challenges in this study as well as in our prior work. These challenges are ubiquitous in our universal health care system and appear to be amplified when clinicians attempt to implement a new treatment program. The context of FBT may bring unique implementation challenges because families must enter treatment urgently. The principles of FBT hold that the young person with AN is at imminent risk and requires parents to act quickly to refeed and to receive treatment rapidly upon referral. This urgency places additional strain on programs already stretched to their limits.

This study also highlights the challenges posed by research-related tasks within the clinical practice context, such as the time required to fax and transfer files electronically, which is burdensome for busy clinicians. In cases where EBTs and associated fidelity supports are implemented in a clinical program outside of research, these elements are not significant.

The selection of clinical cases suited to FBT emerged as a common and significant concern that has been identified previously in the literature [2]. Therapists were reluctant to enrol cases where they felt there was too much patient complexity or co-morbidity, or where parental psychopathology was prominent. They were challenged in maintaining fidelity to the FBT model in cases where self-harm or significant anxiety were present, and subsequently struggled to stay true to all FBT elements. At times, they added components of other evidence-based interventions, such as cognitive behavioural strategies for youth, and emotion focused coaching for parents.

The importance of team communication and collaboration have been identified as paramount in eating disorders treatment to date in order to provide a consistent message to patients and families. We were pleasantly surprised with the results indicating that the medical practitioners were cautious to be involved in any nutritional advice to patients and families, carefully following the protocol in the FBT manual. They felt it was important to leave nutritional decisions for discussion between the therapist and family. This deference to the therapist is important because of how nutritional advice is handled in FBT, which is to give control over nutrition to the parents without dietician involvement in order to empower them to make these decisions. A fundamental principle of the FBT model is that parents are capable of refeeding their child. The objective of the FBT therapist is to support parents to determine *how* to get their child to eat, rather than focusing on *what* their child will eat. For this reason, nutritional decisions are best left to parents who know their child best.

Collegial alliance between therapist and medical practitioner may be an active ingredient in FBT success. A prior study has shown that low collegial alliance as reported by FBT therapists is related to higher rates of dropout from FBT [29]. In addition, collegial alliance reported at session five predicted global EDE-Q scores at end of treatment [29]. Hughes et al. [30] has also reported on the critical nature of collegial alliance between medical staff and therapists when they implemented FBT within their program. Our results resonate with these findings.

A positive impact on clinical services was mentioned by a number of participants, including decreased length of hospital stay and reduced admission rates. This has previously been mentioned by researchers in other parts of the world where FBT has been successfully implemented [31]. It would be prudent for future research to evaluate the extent to which these anecdotal reflections of the impact of the implementation model on hospitalization and admission rates are empirically realized.

The limitations of this study include the small sample size of practitioners and number of sites. Although only twelve individuals participated from four sites, we had representation from small and large health care centres, and our sample represented a range of therapist, medical practitioner and administrator experience. Medical practitioner attendance on implementation calls was under 50%, which may have impacted the frequency of themes attributed to this group. The relatively poor attendance by the medical practitioners might be related to a lack of remuneration for time spent on such calls, as most physicians bill in a fee-for-service manner. This under-representation of this group may not have fully captured their perspectives on the implementation of FBT. The calls themselves may have had an impact on the themes identified as the researchers prompted for difficulties with recruitment and with research implementation if it appeared certain sites had not enrolled patients or had not sent electronic files. The content of the themes also varied with time, with recruitment issues arising upfront. However, several themes were consistent throughout the study including research implementation and communication themes. Although the current findings do not report on treatment fidelity and how it relates to implementation success, this will be reported in a forthcoming paper.

## Conclusions

In summary, this study captures several significant themes brought forward by implementation teams when implementing FBT with fidelity in their clinical programs. Team communication, as well as to role clarity, especially with respect to the therapist and medical practitioner appear to be significant factors in the process of implementation. Barriers to FBT implementation include the characteristics of health care recipients, namely the complexity of some cases and their families, as well as system barriers such as wait lists and resource availability. Many teams mentioned a positive program impact related to the implementation of FBT, therefore further work on addressing system barriers is essential.

Our findings lend support for a number of key recommendations for programs attempting to implement FBT within their clinical services. Programs should financially support time for clinicians, including medical practitioners to communicate fulsomely about outpatients involved in this treatment, including time spent on the specific roles of the individuals involved in the treatment on a weekly basis. Financial support should also be provided for proper triage systems so that individuals with AN are not waiting for lengthy periods on a central intake list. There is a need for specific protocols for identifying and fast-tracking young people with AN so that they are urgently referred for FBT. Similarly, there must be proper resources so that a clinician is available to start working with a family within the FBT model on an urgent basis. With this infrastructure in place, our findings suggest a positive program impact of FBT implementation.

## Abbreviations

AIF: Active Implementation Frameworks
AN: Anorexia Nervosa
BN: Bulimia Nervosa
EBP: Evidence Based Practice
EBT: Evidence Based Treatment
FBT: Family-Based Treatment
